# PftetQ and pfmdt copy numbers as predictive molecular markers of decreased ex vivo doxycycline susceptibility in imported Plasmodium falciparum malaria

**DOI:** 10.1186/1475-2875-12-414

**Published:** 2013-11-14

**Authors:** Tiphaine Gaillard, Sébastien Briolant, Sandrine Houzé, Meïli Baragatti, Nathalie Wurtz, Véronique Hubert, Morgane Lavina, Aurélie Pascual, Christelle Travaillé, Jacques Le Bras, Bruno Pradines

**Affiliations:** 1 Unité de Parasitologie, Institut de Recherche Biomédicale des Armées, Marseille, France; 2 Unité de Recherche sur les Maladies Infectieuses et Tropicales Emergentes, UM 63, CNRS 7278, IRD 198, Inserm 1095, Aix Marseille Université, Marseille, France; 3 Fédération des Laboratoires, Hôpital d’Instruction des Armées Saint Anne, Toulon, France; 4 Laboratoire de Parasitologie-Mycologie, Hôpital Bichat-Claude Bernard, Paris, France; 5 Unité Mixte de Recherche 216 IRD, Université Paris Descartes, Paris, France; 6 Centre National de Référence du Paludisme, Paris, France; 7 Unité de Recherche Mixte Sup-Agro-Inra MISTEA, SupAgro, Montpellier, France; 8 Unité de Recherche Mixte MD3, Institut de Recherche Biomédicale des Armées, Marseille, France

**Keywords:** Malaria, *Plasmodium falciparum*, Anti-malarial, *In vitro*, Resistance, Molecular marker, Doxycycline

## Abstract

**Background:**

The objective of this study was to evaluate the distribution of a series of independent doxycycline inhibitory concentration 50% (IC_50_) values to validate the trimodal distribution previously described and to validate the use of the *pftetQ* and *pfmdt* genes as molecular markers of decreased *in vitro* doxycycline susceptibility in *Plasmodium falciparum* malaria.

**Methods:**

Doxycycline IC_50_ values, from 484 isolates obtained at the French National Reference Centre for Imported Malaria (Paris) between January 2006 and December 2010, were analysed for the first time by a Bayesian mixture modelling approach to distinguish the different *in vitro* phenotypic groups by their IC_50_ values. Quantitative real-time polymerase chain reaction was used to evaluate the *pftetQ* and *pfmdt* copy numbers of 89 African *P. falciparum* isolates that were randomly chosen from the phenotypic groups.

**Results:**

The existence of at least three doxycycline phenotypes was demonstrated. The mean doxycycline IC_50_ was significantly higher in the group with a *pftetQ* copy number >1 compared to the group with a *pftetQ* copy number = 1 (33.17 μM *versus* 17.23 μM) and the group with a *pfmdt* copy number >1 (28.28 μM *versus* 16.11 μM). There was a significant difference between the combined low and medium doxycycline IC_50_ group and the high IC_50_ group in terms of the per cent of isolates with one or more copy numbers of the *pftetQ* gene (0% *versus* 20.69%) or *pfmdt* gene (8.33% *versus* 37.93%). In the logistic regression model, the *pfmdt* and *pftetQ* copy numbers >1 (odds ratio = 4.65 and 11.47) were independently associated with the high IC_50_ group.

**Conclusions:**

Copy numbers of *pftetQ* and *pfmdt* are potential predictive molecular markers of decreased susceptibility to doxycycline.

## Background

Daily administration of doxycycline is currently a recommended chemoprophylactic regimen for travellers visiting malaria-endemic areas with high prevalence of chloroquine or multidrug resistance [1]. In addition, the French malaria consensus recommends quinine and doxycycline for the first-line treatment of *Plasmodium falciparum* severe malaria in Asia and South America. In combination with artesunate or quinine, doxycycline remains the recommendation as the second-line treatment of uncomplicated falciparum malaria or for the treatment of severe malaria as a seven-day course [2]; however, its use is limited. Prophylactic failure of doxycycline against *P. falciparum* has been associated with both inadequate doses [3] and poor compliance [4].

Since September 2002, French troops have participated in the peace-keeping operation, Operation Licorne, in the Ivory Coast. Soldiers had been prescribed doxycycline (100 mg) daily for prophylaxis. Many cases of malaria have been reported, but most of these cases are believed to be the result of poor compliance [5,6]. From 2002 to 2006, 1,787 falciparum malaria cases were observed in French soldiers who were expected to take doxycycline. A surge in the number of malaria cases within three weeks after doxycycline prophylaxis discontinuation is often observed after return [7,8]. Therefore, it is recommended that doxycycline be taken for four weeks after returning from an endemic area. However, resistance can also explain failures of prophylactic doxycycline.

The ability to maximize the efficacy and longevity of anti-malarial drugs for malaria control will depend critically on intensive research to identify *in vitro* markers along with ex vivo and *in vivo* surveillance programmes. It is necessary to identify molecular markers that predict doxycycline resistance or decreased susceptibility in order that active surveillance can monitor temporal trends in parasite susceptibility [9]. Although there have been no reported clinical failures for the treatment of falciparum malaria with doxycycline, a Bayesian mixture modelling approach has distinguished three different *in vitro* phenotypic groups: low, medium and high doxycycline IC_50_ values, among 747 *P. falciparum* isolates obtained from 14 African countries over a nine-year period [10]. The sequences of 11 *P. falciparum* genes that are analogous to those involved in bacterial resistance to doxycycline were obtained from 30 isolates from each phenotypic group. The data suggested that the copy numbers of a *tet*Q GTPase family gene, *pftetQ* (PFL1710c), and a metabolic drug transporter gene, *pfmdt* (PFE0825w), were potential molecular markers of decreased *in vitro* susceptibility to doxycycline in African isolates [11].

The objective of this study was first to evaluate the distribution of a new series of independent doxycycline IC_50_ values assessed by another group for goodness of fit with the trimodal compartment model of doxycycline response previously proposed [10] and then to validate the use of the *pftetQ* and *pfmdt* genes as molecular markers of decreased *in vitro* susceptibility to doxycycline. This was performed by assessing the gene copy numbers in *P. falciparum* clinical isolates that were randomly chosen from the phenotypic groups with different doxycycline IC_50_ values.

## Methods

### Patients and sample collection

Between January 2006 and December 2010, 484 fresh *P. falciparum* isolates were obtained at the French National Reference Centre for Imported Malaria (Paris) from patients hospitalized with malaria after having returned to France. These samples were successfully assessed for doxycycline susceptibility. Ex vivo testing of doxycycline susceptibility was performed as previously described by a standard 42-hour ^3^H-hypoxanthine uptake inhibition assay [12]. Batches of plates were tested and validated on the chloroquine-susceptible 3D7 strain and the chloroquine-resistant W2 strain.

The drug concentration that inhibited 50% parasite growth (IC_50_) was calculated with the inhibitory sigmoid Emax model, with estimation of the IC_50_ through non-linear regression using a standard function of the R software (ICEstimator) [13].

### Quantification of pftetQ and pfmdt copy numbers

*pfmdt* (PFE0825w) and *pftetQ* (PFL1710c) copy numbers were estimated by TaqMan real-time PCR (7900HT Fast Real-Time PCR system, Applied Biosystems) relative to the single-copy gene, *pfβtubulin* (PF10_0084). The following oligonucleotide primers and probes were designed using the Primer Express software v2.0 (Applied Biosystems) for use in the polymerase chain reactions (PCRs): 5′-TTATGCAAACATTTCAAGCTTCCT-3′, 5′- ACCCATTCCATAACTTAGATTTAGATAACC-3′ and 5′-VIC-TAAAAACAAATTTCGACAAAAGGACAGGAGCC-TAMRA-3′ for *pfmdt*, 5′-ACCCCTTTTTTATCTTACGAAAG-3′, 5′-ATGGTTGTACGTTATATCATATGG-3′ and 5′-VIC-AAAAATGTGGCAACAATTCAGACATGTATCA-TAMRA-3′ for *pftetQ* and 5′-TGATGTGCGCAAGTGATCC-3′, 5′-TCCTTTGTG GACATTCTTCCTC-3′ and 5′-FAM-TAGCACATGCCGTTAAATATCTTCCATGTCT-TAMRA-3′ for *pfβtubulin* (Eurogentec). Individual PCRs were performed using 1 X TaqMan Universal PCR Master Mix (Applied Biosystems), 900 nM forward primer, 900 nM reverse primer, 250 nM TaqMan probe and 5 μL template DNA in a final volume of 25 μL The reaction mixtures were prepared at 4°C in a 96-well optical reaction plate (Applied Biosystems) covered with optical adhesive covers (Applied Biosystems). The thermal cycling conditions were 50°C for 2 min, 95°C for 10 min and 50 cycles of 95°C for 15 sec and 60°C for 1 min. Each sample was assayed in triplicate and analysed with the SDS software 2.2.1 (Applied Biosystems). The PCR efficiencies of all the primer pairs were evaluated on a dilution series of *P. falciparum* 3D7 genomic DNA. The efficiencies were found to be sufficiently close to obviate the need for any correction factor. Therefore, the 2^-ΔΔCt^ method of relative quantification was used and adapted to estimate the number of copies of the *pfmdt* and *pftetQ* genes [14,15] with the formula ΔΔCt = (Ct_*pfmdt*_ - Ct_*pfβtubulin*_) _sample_ - (Ct_*pfmdt*_ – Ct_*pfβtubulin*_) _calibrator_. Genomic DNA extracted from 3D7 *P. falciparum*, which has a single copy of each gene, was used for calibration, whereas *pfβtubulin* served as the control housekeeping gene in all the experiments.

### Genetic diversity of Plasmodium falciparum isolates with pftetQ and pfmdt multicopies

Mixed infection could influence read-out in Taqman real-time PCR, potentially leading to false positive results of gene copy number. The genomic DNA of *P. falciparum* isolates with at least two copies of *pfmdt* or *pftetQ* were investigated for genetic diversity at highly polymorphic loci, merozoite surface proteins 1 and 2 (MSP1 and MSP2). The *msp1* and *msp2* loci were genotyped using the nested PCR strategy and conditions previously described [16].

### Statistical analysis

The statistical analysis has been designed to answer the specific question of whether *P. falciparum* has different doxycycline susceptibility phenotypes. A heterogeneous population of IC_50_ values was observed; therefore, the data were assumed to represent a univariate Gaussian mixture with k components. Each observation was assumed to originate from one of the k components, and the label of the group from which each observation arose was unknown. The unknowns of the model were the number of components, the means, variances and weights of the different components, and the vector of allocations of the observations. The analysis was performed in two steps. First, reversible jump Monte Carlo Markov Chains (RJMCMC) [17] samplers were used to choose a suitable number of components k, and the present algorithm followed the recommendations of Cappé et al. [18]. After a relevant number of components was chosen, standard Gibbs samplers were run to obtain estimates of the model parameters and to classify the observations [19]. Because of the’label-switching’ problem, due to the symmetry in the likelihood of the model parameters, the mixture components should be labelled before making an inference on the parameters [20]. The classical ordering constraint, which was biologically relevant here, was used. The algorithms were run for 5,000 burn-in iterations and 20,000 post-burn-in iterations. These numbers were assumed to be sufficient to obtain reliable results. Moreover, each algorithm was run three times to check that the results between two different runs were similar and that there was no convergence problem [17].

The data were analysed using the R software® (version 2.10.1). The differences in the *pfmdt* and *pftetQ* copy numbers between the phenotypic groups were tested using the Mann Whitney test and the Kruskal-Wallis test. The genotype proportions were compared using the Fisher exact test. The risk of the high doxycycline IC_50_ was analysed using a logistic regression model (univariate and multivariate analysis).

### Ethics

Informed consent was not required for this study because the sampling procedures and testing are part of the French national recommendations for the care and surveillance of malaria.

## Results

The doxycycline IC_50_ values ranged from 0.49 to 65.1 μM. The mean was 11.64 μM (95% confidence interval, 10.96-12.33). The average parameter estimates for the IC_50_ values by year are given in Table 1.

**Table 1:** **Statistical analysis of the 484 doxycycline IC**_**50 **_**values by year**

**Year**	**IC**_**50 **_**number**	**Mean (μM)**	**95% CI**	**IC**_**50 **_**min**	**IC**_**50 **_**max**
2006	119	10.05	9.14-10.96	0.63	43.7
2007	172	11.91	10.72-13.11	2.34	44.8
2008	59	9.45	8.56-10.35	4.27	23.1
2009	40	12.21	8.01-16.41	0.49	65.1
2010	94	14.3	12.63-15.97	4.55	46.2
Total	484	11.64	10.96-12.33	0.49	65.1

95% CI: 95% confidence interval.

**Figure 1:**
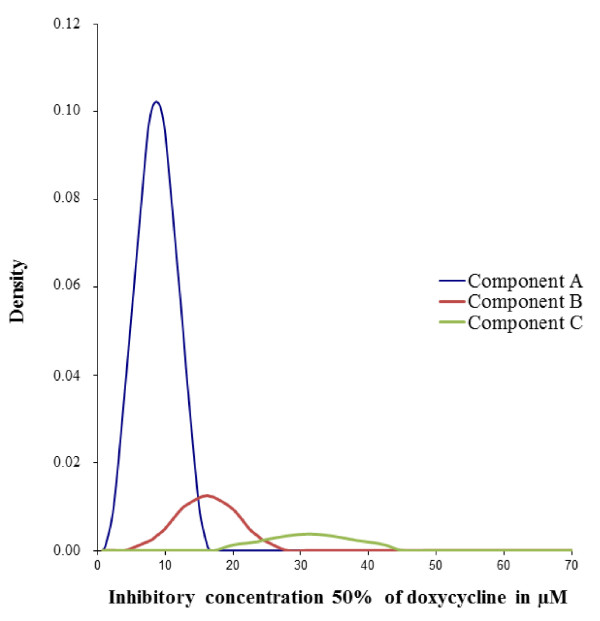
**Distribution of the doxycycline IC**_**50 **_**values of the 484 *Plasmodium falciparum *isolates (2006 to 2010) in the three-component mixture model (Bayesian mixture modelling approach).** The dotted lines represent the three fitted mixtures.

The triple normal distribution model is represented in Figure 1. The parameter estimates for the three-component mixture model, including the number of isolates in each normal distribution, the mean of the IC_50_ values and the standard deviation for each distribution, are summarised in Table 2. A double normal distribution model and a quadruple normal distribution model were also fitted to the data to assess the validity of considering a three-component mixture (data not shown). These two models fit the data worse than the triple normal distribution model.

**Table 2:** **Parameter estimates for the three-component mixture model for the 484 *Plasmodium falciparum *** **isolates**

**Component**	**Isolates number**	**Proportion (%)**	**IC**_**50 **_**mean (μM)**	**Standard deviation**
A	393	81.1	8.70	2.87
B	60	12.4	16.18	4.86
C	31	6.5	31.23	10.32

Eighty-nine *P. falciparum* isolates (30, 30 and 29) were randomly chosen from the three phenotypic groups, A, B and C, that differed in their doxycycline IC_50_ values. These isolates were classified as follows: low doxycycline IC_50_ group from component A [mean, 4.33 μM (95% CI, 3.39-4.37 μM)], medium doxycycline IC_50_ group from component B [mean, 16.97 μM (95% CI, 16.45-17.49 μM)] and high doxycycline IC_50_ group from component C [mean, 34.60 μM (95% CI, 31.30-37.90 μM)].

Only one or two copies of *pfmdt* and *pftetQ* were identified in the 89 isolates. All of the isolates with two copies *pfmdt* or *pftetQ* had 1 allelic family for each of the two genes (*msp1* and *msp2*), confirming that these infections were single and not mixed. The mean doxycycline IC_50_ was significantly higher in the group with a *pftetQ* copy number >1 compared to the group with a *pftetQ* copy number = 1 (33.17 μM *versus* 17.23 μM; *P* = 0.0041, Mann Whitney test) (Table 3). The mean doxycycline IC_50_ was significantly higher in the group with a *pfmdt* copy number >1 (28.28 μM *versus* 16.11 μM; *P* = 0.0025, Mann–Whitney test).

**Table 3:** **Statistical analysis of the doxycycline IC**_**50 **_**values based on the *pftetQ *** **and *pfmdt *** **copy numbers in 89 *Plasmodium falciparum *** **isolates**

	***pftetQ *** **copy number**		***pfmdt *** **copy number**	
	**= 1**	**> 1**	**= 1**	**> 1**
Number of values	83	6	73	16
IC_50_ mean (μM)	17.23	33.17	16.11	28.28
Standard deviation	13.32	6.85	12.53	14.03
95% Confidence interval	14.32-20.13	25.98-40.37	13.19-19.04	20.81-35.76
Minimal IC_50_	0.49	25.16	0.49	4.62
Maximal IC_50_	65.11	43.7	44.82	65.11

The number of *pftetQ* copies was significantly higher in the high doxycycline IC_50_ group compared to the low and medium doxycycline IC_50_ groups (1.21 *versus* 1.0 and 1.0; *P* = 0.0014, Kruskal-Wallis test). The number of *pfmdt* copies was significantly higher in the high doxycycline IC_50_ group compared to the low and medium doxycycline IC_50_ groups (1.38 *versus* 1.13 and 1.03, respectively; *P* = 0.0019, Kruskal-Wallis test).

There was no significant difference between the low and medium doxycycline IC_50_ groups for the *pfmdt* and *pftetQ* copy numbers. Therefore, these two phenotypic groups were combined. There was a statistically significant difference between the low and medium doxycycline IC_50_ combined group and the high doxycycline group in terms of the per cent of isolates with one or more copy numbers of the *pftetQ* gene (0% *versus* 20.69%; *P* = 0.0008, Fisher’s exact test) or *pfmdt* gene (8.33% *versus* 37.93%; *P* = 0.0021, Fisher’s exact test) (Table 4).

**Table 4:** **Statistical analysis of *pftetQ *** **and *pfmdt *** **copy numbers in 89 *Plasmodium falciparum *** **isolates (Fisher’s exact test)**

	***PftetQ***		***Pfmdt***	
	**Low and medium IC**_**50**_	**High IC**_**50**_	**Low and medium IC**_**50**_	**High IC**_**50**_
Copy number >1	0	6	5	11
Copy number = 1	60	23	55	18
%	0.00	20.69	8.33	37.93
Fisher’s exact test	*P* value	0.0008	*P* value	0.0021

In the logistic regression model (Table 5), the *pfmdt* copy number >1 (adjusted OR = 4.65 [1.31-16.51], *P* = 0.0176) and *pftetQ* copy number >1 (adjusted OR = 11.47 [1.23-106.98], *P* = 0.0322) were independently associated with the high IC_50_ phenotypic group.

**Table 5:** Multivariate regression model

	**Doxycycline IC**_**50 **_**group, number**					
**Molecular marker**	**Low or medium**	**High**	**Crude OR (95% CI)**	***P***	**Adjusted OR (95% CI)**	***P***
*pftetQ* copy number						
1	60	23	1.00 (reference)		1.00 (reference)	
> 1	0	6	18.77 (2.18-161.43)	0.0076	11.47 (1.23-106.98)	0.0322
*pfmdt* copy number						
1	55	5	1.00 (reference)		1.00 (reference)	
> 1	18	11	6.72 (2.06-21.96)	0.0016	4.65 (1.31-16.51)	0.0176

## Discussion

Most prophylactic failures of doxycycline against *P. falciparum* are associated with the use of standard doses resulting in lower than expected serum drug levels [21], inadequate low doses [3], or poor compliance [4,22]. Moreover, doxycycline pharmacokinetic parameters could explain some of these cases. Doxycycline has a short elimination half-life (16 hours) compared to proguanil (24 hours), atovaquone (31–73 hours), chloroquine (two to three days), or mefloquine (six to 41 days), and a short mean residence time (63% of the administered dose is eliminated in 27 hours) [8]. In addition, its slow action *in vitro* has a delayed effect upon growth and requires the prolonged incubation of parasites [23]. Determination of the IC_50_ after two generations of parasite growth decreases the 42-hour IC_50_ from ten- to 20-fold [24,25]. However, in practice, the standard 42-hour test remains the method of monitoring doxycycline ex vivo susceptibility.

Maximizing the efficacy and longevity of anti-malarial drugs to control malaria will critically depend on intensive research to identify *in vitro* markers along with the implementation of ex vivo and *in vivo* surveillance programmes, such as those championed by the WorldWide Antimalarial Resistance Network [26]. Therefore, there is a need to identify molecular markers that predict doxycycline resistance, which can provide an active surveillance method to monitor temporal trends in parasite susceptibility [9]. In addition, the early detection of resistance or decreased susceptibility to doxycycline will require that the baseline parasite chemosusceptibility of current isolates from endemic regions is established.

To validate the trimodal distribution model of doxycycline IC_50_ values previously described for *P. falciparum* African isolates [10], the distribution of a new series of independent doxycycline IC_50_ values that were assessed by a separate group under the same technical conditions [12] was evaluated. This analysis was performed with a Bayesian mixture modelling approach. Again, the demonstration of the existence of at least three doxycycline phenotypes was confirmed. All 484 values were classified into three components: component A (IC_50_ mean 8.7 μM), component B (IC_50_ mean 16.2 μM), and component C (IC_50_ mean 31.2 μM). This trimodal distribution model of doxycycline IC_50_ values from imported *P. falciparum* isolates obtained from 2006 to 2010 confirms the previous data [10]. However, the level of the IC_50_ value in each component is different between the series of imported *P. falciparum* isolates obtained from 2006 to 2010 and those obtained from 1999 to 2006 [10]. The IC_50_ doxycycline values from the isolates obtained from 1999 to 2006 were classified into three components: component A (IC_50_ mean 4.9 μM), component B (IC_50_ mean 7.7 μM), and component C (IC_50_ mean 17.9 μM). It appears that components A and B (IC_50_ means 4.9 μM and 7.7 μM, respectively) for the values obtained from 1999 to 2006 have merged into a single component A (IC_50_ mean 8.7 μM) for the values obtained from 2006 to 2010. In addition, the percentage of isolates (78%) in the two components, A and B, for the values obtained from 1999 to 2006 is similar to the percentage of isolates (81%) for component A for the values obtained from 2006 to 2010. The component B (IC_50_ mean 16.2 μM) values obtained from 2006 to 2010 correspond to the component C (IC_50_ mean 17.9 μM) values obtained from 1999 to 2006. A new component, component C (IC_50_ mean 31.2 μM, proportion 6.4%), emerged for the values obtained from 2006 to 2010. These data suggest the emergence of strains with decreased susceptibility to doxycycline. In addition, based on the previously defined cut-off for reduced susceptibility to doxycycline (35 μM) [10], 1.2% of the 747 *P. falciparum* isolates tested from 1999 to 2006 were considered to have decreased susceptibility to doxycycline *versus* 2.7% for the 484 isolates tested from 2006 to 2010.

*Plasmodium falciparum* possesses a *tetQ* GTPase family gene analogue of the genes that encode bacterial ribosomal protection proteins. These genes are the *pftetQ,* which is involved in bacterial resistance to the cycline drugs, and a multidrug transporter gene, *pfmdt*, which shares a high sequence identity with efflux pumps. In a multivariate logistic regression model, an increased *pfmdt* copy number was associated with high doxycycline IC_50_ values with an adjusted odds ratio (OR) of 7.09 (p = 0.011), and an increased *pftetQ* copy number was associated with an adjusted OR of 5.23 (p = 0.042) [11]. To validate the use of the *pftetQ* and *pfmdt* genes as molecular markers of decreased *in vitro* susceptibility to doxycycline by assessing the gene copy numbers, 89 (30, 30 and 29) *P. falciparum* clinical isolates were randomly chosen from the three phenotypic groups (A, B and C) with different doxycycline IC_50_ values. These isolates were classified as follows: low doxycycline IC_50_ group from component A [mean, 4.33 μM (95% CI, 3.39-4.37 μM)], medium doxycycline IC_50_ group from component B [mean, 16.97 μM (95% CI, 16.45-17.49 μM)] and high doxycycline IC_50_ group from component C [mean, 34.60 μM (95% CI, 31.30-37.90 μM)]. These isolates were obtained from patients hospitalized with malaria after travel in Cameroon (n = 18), Ivory Coast (n = 14), Mali (n = 11), Niger (n = 5), Burundi (n = 4), Burkina Faso (n = 4), Djibouti (n = 4), Madagascar (n = 4), Congo (n = 5), Ghana (n = 3), Sudan (n = 2), Central African Republic (n = 2), Zambia (n = 1), Rwanda (n = 1), Togo (n = 1), Guinea (n = 1), and nine from Africa without specificity regarding the country.

The mean doxycycline IC_50_ value is significantly higher in the groups with *pftetQ* or *pfmdt* copy numbers >1, suggesting that *pftetQ* and *pfmdt* could be involved in the reduced susceptibility to doxycycline.

The number of *pftetQ* and *pfmdt* gene copies is significantly higher in the high doxycycline IC_50_ group than the low and medium doxycycline IC_50_ groups. However, there is no significant difference between the low and the medium doxycycline IC_50_ groups for the *pfmdt* and *pftetQ* copy numbers. These two phenotypic groups were, therefore, combined. There is a statistically significant difference between the low and medium doxycycline IC_50_ combined group and the high doxycycline group in terms of the per cent of isolates with one or more copy numbers of the *pftetQ* gene (0% *versus* 20.69%; *P* = 0.0008) or *pfmdt* gene (8.33% *versus* 37.93%; *P* = 0.0021). In addition, in the multivariate logistic regression model, an increased *pfmdt* copy number is associated with high doxycycline IC_50_ values with an adjusted OR of 4.65 (*P* = 0.0176), and an increased *pftetQ* copy number is associated with an adjusted OR of 11.47 (*P* = 0.0322). These results are consistent with previous data [11] and confirm the potential use of *pftetQ* and *pfmdt* as predictive molecular markers for decreased *P. falciparum* susceptibility to doxycycline in Africa.

In a study on fresh *P. falciparum* clinical isolates from Dakar, Senegal, it was shown that there was no statistically significant difference between a group with a doxycycline IC_50_ <25 μM and a group with an IC_50_ >25 μM in terms of the per cent of isolates with one or more copy numbers of the *pftetQ* gene (p = 0.079) or *pfmdt* gene (p = 0.066) [27]. However, the significance levels of these associations were just above the *P* value threshold (0.05). It seems that the number of isolates from the high doxycycline IC_50_ group (15.9%) was most likely too low to obtain statistically significant differences, indicating the necessity of assessing the gene copy numbers with more isolates. Another possibility is that over-expression of *pftetQ* or *pfmdt* could confer *in vitro* reduced susceptibility to doxycycline in association with other contributing determinants, which could modulate the *in vitro* response to doxycycline.

In summary, this study demonstrates that copy numbers of the *pftetQ* and *pfmdt* genes are potential predictive molecular markers of decreased *P. falciparum* susceptibility to doxycycline in Africa. Epidemiological studies using large numbers of parasites with reduced susceptibility to doxycycline are now required to determine whether *pftetQ* and *pfmdt* can be used as markers of reduced *in vitro* doxycycline susceptibility.

## Competing interests

The authors have declared that they have no competing interests.

## Authors’ contributions

TG, NW, ML and AP carried out the molecular genetic studies. SH, VH and JLB carried out the ex vivo evaluation of doxycycline susceptibility. The French National Reference Centre for Imported Malaria Study Group supervised, carried out and coordinated the field collections of patient isolates. BP and SB conceived and coordinated the study. SB, MB, CT and BP analysed the data. TG, SB, SH, MB, JLB and BP drafted the manuscript. All the authors read and approved the final manuscript.

## Authors’ information

French National Reference Centre for Imported Malaria Study Group: A Aboubacar (CHU Strasbourg), Agnamey P. (CHU Amiens), Ajana F. (CH Tourcoing), Amal C. (CH Mourier, Colombes), Amira R. (CHG Saint Denis), Argy N. (CHU Bichat-Claude Bernard, Paris), Baumard S. (CHRU Reims), Bellanger A. P. (CHU Minjoz, Besancon), Bemba D. (CH Verdier, Bondy), Beytout J. (CHRU Clermont Ferrand), Bigel M.L. (CH Quesnay, Mante la Jolie), Bloch M. (CH Mourier, Colombes), Bonnet R. (CHRU Clermont Ferrand), Borel A. (CHU Amiens), Bouchaud O. (CH Avicenne, Bobigny), Branger C. (CH Mourier, Colombes), Bruneel F. (CH Mignot, Versailles), Cambon M. (CHRU Clermont Ferrand), Camus D. (CH Lille), Casalino E. (CHU Bichat-Claude Bernard, Paris), Clain J. (CHU Bichat-Claude Bernard, Paris), Cojean S. (CHU Bichat-Claude Bernard, Paris), Cuisenier B. (CHU Dijon), De Gentile L. (CHU Angers), Delarbre J. M. (CH Moenchsberg, Mulhouse), Delaval A. (CH Balanger, Aulnay sous Bois), Durand R. (CH Avicenne, Bobigny), Dutoit E. (CH Lille), Eloy O. (CH Mignot, Versailles), Faucher J. F. (CHU Minjoz, Besancon), Faye A. (CH Debre, Paris), Fenneteau O. (CH Debre, Paris), Filisetti D. (CHU Strasbourg), Fulleda C. (CHU Lariboisiere, Paris), Godineau N. (CHG Saint Denis), Grenouillet F. (CHU Minjoz, Besancon), Hurst J. P. (CH Monod, Le Havre), Ichou H. (CH Mourier, Colombes), Klein E. (CHU Lariboisiere, Paris), Lariven S. (CHU Bichat-Claude Bernard, Paris), Lefevre M. (CH Laennec, Creil), Lemoine M. (CHU Bichat-Claude Bernard, Paris), Lesens O. (CHRU Clermont Ferrand), Lohmann C. (CH Moenchsberg, Mulhouse), Lusina D. (CH Balanger, Aulnay sous Bois), Machouart M. C. (CHR Nancy), Mary R. (CHG Saint Denis), Matheron S. (CHU Bichat-Claude Bernard, Paris), Mechali D. (CHG Saint Denis), Merrens A. (HIA Begin, Saint Mande), Millon L. (CHU Minjoz, Besancon), Monnier S. (CH Mignot, Versailles), Mortier E. (CH Mourier, Colombes), Moussel F. (CH Quesnay, Mante la Jolie), Pageot L. (CHU Bichat-Claude Bernard, Paris), Parez N. (CH Mourier, Colombes), Patoz P. (CH Tourcoing), Pfaff A. (CHU Strasbourg), Pihet M. (CHU Angers), Pilo J. E. (HIA Begin, Saint Mande), Poilane I. (CH Verdier, Bondy), Pons D. (CHRU Clermont Ferrand), Poupart M. (CHG Saint Denis), Prevel M. (CHG Saint Denis), Pull L. (CH Debre, Paris), Rapp C. (HIA Begin, Saint Mande), Rivier A. (CHR Nancy), Ronez E. (CHU Lariboisiere, Paris), Rotten D. (CHG Saint Denis), Sarrasin V. (CHU Bichat-Claude Bernard, Paris), Silva M. (CH Monod, Le Havre), Simonet A. L. (CHU Dijon), Siriez J. Y. (CH Debre, Paris), Strady C. (CH Debre, Reims), Therby A. (CH Mignot, Versailles), Thibault M. (CH Dubos, Cergy Pontoise), Thouvenin M. (CH Troyes), Toubas D. (CHRU Reims).

## References

[B1] Société de Pathologie Infectieuse de Langue Française, Collège des Universitaires de Maladies Infectieuses et Tropicales, Société de Médecine des Armées, Société Française de Parasitologie, Société Française de Pédiatrie, Société de Médecine des Voyages, Société de Pathologie Exotique, Société de Réanimation de Langue Française Management and prevention of imported *Plasmodium falciparum* malaria: recommendations for clinical practice 2007 (revision 2007 of the 1999 consensus conference) Med Mal Infect 2008 38 68 117 18646360 10.1016/j.medmal.2007.11.007

[B2] World Health Organization WHO guidelines for the treatment of malaria 2010 2 Geneva: WHO Press 25473692

[B3] Pang L Limsomwong N Singharaj P Prophylactic treatment of vivax and falciparum malaria with low-dose doxycycline J Infect Dis 1988 158 1124 1127 10.1093/infdis/158.5.1124 3053925

[B4] Wallace MR Sharp TW Smoak B Iriye C Rozmajzl P Thornton SA Batchelor R Magill AJ Lobel HO Longer CF Burans JP Malaria among United States troops in Somalia Am J Med 1996 100 49 55 10.1016/S0002-9343(96)90011-X 8579087

[B5] Migliani R Josse R Hovette R Keundjian A Pages F Meynard JB Ollivier L Sbai Idrissi K Tifratene K Orlandi E Rogier C Boutin JP Le paludisme vu des tranchées: le cas de la Côte d’Ivoire en 2002–2003 Med Trop 2003 63 282 286 14579467

[B6] Migliani R Ollivier L Romand O Verret C Haus-Cheymol R Todesco A Pagès F Pradines B Queyriaux B Texier G Michel R Spiegel A Boutin JP Paludisme chez les militaires français en Côte d’Ivoire de 1998 à 2006 Bull Epidemiol Hebdom 2008 23–24 209 212

[B7] Pang LW Limsomwong N Boudreau EF Singharaj P Doxycycline prophylaxis for falciparum malaria Lancet 1987 i 1161 1164 2883488 10.1016/s0140-6736(87)92141-6

[B8] Shmuklarsky MJ Boudreau EF Pang LW Smith JI Schneider I Fleckenstein L Abdelrahim MM Canfield CJ Schuster B Failure of doxycycline as a causal prophylactic agent against Plasmodium falciparum malaria in healthy nonimmune volunteers Ann Int Med 1994 120 294 299 10.7326/0003-4819-120-4-199402150-00006 8291822

[B9] Plowe CV Roper C Barnwell JW Happi CT Joshi HH Mbacham W Meshnick SR Mugittu K Naidoo I Price RN Shafer RW Sibley CH Sutherland CJ Zimmerman PA Rosenthal PJ World antimalarial resistance network (WARN). III: molecular markers for drug resistant malaria Malar J 2007 6 121 10.1186/1475-2875-6-121 17822535 PMC2008207

[B10] Briolant S Baragatti M Parola P Simon F Tall A Sokhna C Hovette P Mamfoumbi MM Koeck JL Delmont J Spiegel A Castello J Gardair JP Trape JF Kombila M Minodier P Fusai T Rogier C Pradines B Multinormal *in vitro* distribution model suitable for the distribution of Plasmodium falciparum chemosusceptibility to doxycycline Antimicrob Agents Chemother 2009 53 688 695 10.1128/AAC.00546-08 19047651 PMC2630600

[B11] Briolant S Wurtz N Zettor A Rogier C Pradines B Susceptibility of Plasmodium falciparum isolates do doxycycline is associated with pftetQ sequence polymorphisms and pftetQ and pfmdt copy numbers J Infect Dis 2010 201 152 159 10.1086/648594 19929377

[B12] Parola P Pradines B Simon F Carlotti MP Minodier P Ranjeva MP Badiaga S Bertaux L Delmont J Morillon M Silai R Brouqui P Parzy D Antimalarial drug susceptibility and point mutations associated with resistance in 248 Plasmodium falciparum isolates imported from Comoros to Marseille Am J Trop Med Hyg 2007 77 431 437 17827355

[B13] Le Nagard H Vincent C Mentré F Le Bras J Online analysis of *in vitro* resistance to antimalarial drugs through nonlinear regression Comput Methods Programs Biomed 2011 104 10 18 10.1016/j.cmpb.2010.08.003 20828858

[B14] Livak KJ Schmittgen TD Analysis of relative gene expression data using real-time quantitative PCR and the 2(-Delta Delta C(T)) Method Methods 2001 25 402 408 10.1006/meth.2001.1262 11846609

[B15] Ferreira ID Rosario VE Cravo PV Real-time quantitative PCR with SYBR Green I detection for estimating copy numbers of nine drug resistance candidate genes in Plasmodium falciparum Malar J 2006 5 1 10.1186/1475-2875-5-1 16420686 PMC1363351

[B16] Henry M Diallo I Bordes J Ka S Pradines B Diatta B M’Baye PS Sane M Thiam M Gueye PM Wade B Touze JE Debonne JM Rogier C Fusai T Urban malaria in Dakar, Senegal: chemosusceptibility and genetic diversity of Plasmodium falciparum isolates Am J Trop Med Hyg 2006 75 146 151 16837722

[B17] Richardson S Green PJ On Bayesian analysis of mixtures with an unknown number of components (with discussion) J Roy Stat Soc 1997 B59 731 792

[B18] Cappé O Robert CP Rydén T Reversible jump, birth-and-death and more general continuous time Markov chain Monte Carlo samplers J Roy Stat Soc 2003 B65 679 700

[B19] Diebolt J Robert CP Estimation of finite mixture distributions through Bayesian sampling J Roy Stat Soc 1994 56 363 375

[B20] Jasra A Holmes CC Stephens DA Markov chain Monte Carlo methods and the label switching problem in Bayesian mixture modeling Statistical Science 2005 20 50 67 10.1214/088342305000000016

[B21] Weiss WR Oloo AJ Johnson A Koech D Hoffman SL Daily primaquine is effective for prophylaxis against falciparum malaria in Kenya: comparison with mefloquine, doxycycline, and chloroquine plus proguanil J Infect Dis 1995 171 1569 1575 10.1093/infdis/171.6.1569 7769294

[B22] Shanks GD Roessler P Edstein M Rieckmann KH Doxycycline for malaria prophylaxis in Australian soldiers deployed to United Nations missions in Somalia and Cambodia Mil Med 1995 160 443 444 7478027

[B23] Dahl EL Rosenthal PJ Multiple antibiotics exert delayed effects against the Plasmodium falciparum apicoplast Antimicrob Agents Chemother 2007 51 3485 3490 10.1128/AAC.00527-07 17698630 PMC2043295

[B24] Pradines B Spiegel A Rogier C Tall A Mosnier J Fusai T Trape JF Parzy D Antibiotics for prophylaxis of Plasmodium falciparum infections: *in vitro* activity of doxycycline against Senegalese isolates Am J Trop Med Hyg 2000 62 82 85 10761729 10.4269/ajtmh.2000.62.82

[B25] Pradines B Rogier C Fusai T Mosnier J Daries W Barret E Parzy D *In vitro* activities of antibiotics against Plasmodium falciparum are inhibited by iron Antimicrob Agents Chemother 2001 45 1746 1750 10.1128/AAC.45.6.1746-1750.2001 11353621 PMC90541

[B26] Sibley CH Barnes KI Watkins WM Plowe CV A network to monitor antimalarial drug resistance: a plan for moving forward Trends Parasitol 2008 24 43 48 10.1016/j.pt.2007.09.008 18042432

[B27] Gaillard T Fall B Tall A Wurtz N Diatta B Lavina M Fall KB Sarr FD Baret E Diémé Y Wade B Bercion R Briolant S Pradines B Absence of association between ex vivo susceptibility to doxycycline and pftetQ and pfmdt copy numbers in Plasmodium falciparum isolates from Dakar, Senegal Clin Microbiol Infect 2012 18 E238 E240 10.1111/j.1469-0691.2012.03889.x 22533855

